# Analysis of predicted loss-of-function variants in UK Biobank identifies variants protective for disease

**DOI:** 10.1038/s41467-018-03911-8

**Published:** 2018-04-24

**Authors:** Connor A. Emdin, Amit V. Khera, Mark Chaffin, Derek Klarin, Pradeep Natarajan, Krishna Aragam, Mary Haas, Alexander Bick, Seyedeh M. Zekavat, Akihiro Nomura, Diego Ardissino, James G. Wilson, Heribert Schunkert, Ruth McPherson, Hugh Watkins, Roberto Elosua, Matthew J. Bown, Nilesh J. Samani, Usman Baber, Jeanette Erdmann, Namrata Gupta, John Danesh, Daniel Chasman, Paul Ridker, Joshua Denny, Lisa Bastarache, Judith H. Lichtman, Gail D’Onofrio, Jennifer Mattera, John A. Spertus, Wayne H.-H. Sheu, Kent D. Taylor, Bruce M. Psaty, Stephen S. Rich, Wendy Post, Jerome I. Rotter, Yii-Der Ida Chen, Harlan Krumholz, Danish Saleheen, Stacey Gabriel, Sekar Kathiresan

**Affiliations:** 1 Center for Genomic Medicine, Massachusetts General Hospital, Harvard Medical School, Boston, MA 02114 USA; 2 Department of Medicine, Massachusetts General Hospital, Cardiology Division, Harvard Medical School, Boston, MA 02114 USA; 3 Program in Medical and Population Genetics, Broad Institute, Cambridge, MA 02142 USA; 4 Department of Surgery, Massachusetts General Hospital, Harvard Medical School, Boston, MA 02114 USA; 5 Department of Computational Biology & Bioinformatics, Yale Medical School, Yale University, New Haven, MA 06510 USA; 6 Division of Cardiology, Azienda Ospedaliero–Universitaria di Parma, Parma, 43121 Italy; 7 Associazione per lo Studio Della Trombosi in Cardiologia, Pavia, 27100 Italy; 8 Department of Physiology and Biophysics, University of Mississippi Medical Center, Jackson, MS 39216 USA; 9 Deutsches Herzzentrum München, Technische Universität München, Deutsches Zentrum für Herz-Kreislauf-Forschung, München, 80333 Germany; 10 University of Ottawa Heart Institute, Ottawa, ON K1Y4W7 Canada; 11 Radcliffe Department of Medicine, Division of Cardiovascular Medicine, University of Oxford, Oxford, OX1 2JD UK; 12 Wellcome Trust Centre for Human Genetics, University of Oxford, Oxford, OX1 2JD UK; 13 Cardiovascular Epidemiology and Genetics, Hospital del Mar Research Institute, Barcelona, 08003 Spain; 14 CIBER Enfermedades Cardiovasculares (CIBERCV), Barcelona, 28029 Spain; 15 Facultat de Medicina, Universitat de Vic-Central de Cataluña, Barcelona, VIC 08500 Spain; 16 Department of Cardiovascular Sciences, University of Leicester, and NIHR Leicester Biomedical Research Centre, Leicester, LE1 7RH UK; 17 The Zena and Michael A. Wiener Cardiovascular Institute, Icahn School of Medicine at Mount Sinai, New York, 10029 NY USA; 18 Institute for Integrative and Experimental Genomics, University of Lübeck, Lübeck, 23562 Germany; 19 Department of Public Health and Primary Care, Cardiovascular Epidemiology Unit, University of Cambridge, Cambridge, CB2 0SR UK; 20 Wellcome Trust Sanger Institute, Hinxton, Cambridge CB10 1SA UK; 21 National Institute of Health Research Blood and Transplant; Research Unit in Donor Health and Genomics, University of Cambridge, Cambridge, CB2 1TN UK; 22 Center for Cardiovascular Disease Prevention, Brigham and Women’s Hospital, Boston, 02115 USA; 23 Department of Biomedical Informatics, Vanderbilt University, Nashville, TN 37235 USA; 24 Department of Chronic Disease Epidemiology, Yale School of Public Health, New Haven, CT 06510 USA; 25 Department of Emergency Medicine, Yale University, New Haven, CT 06520 USA; 26 Center for Outcomes Research and Evaluation, Yale–New Haven Hospital, New Haven, CT 06510 USA; 27 Department of Biomedical & Saint Luke’s Mid America Heart Institute and the Health Informatics, Division of Endocrinology and Metabolism, University of Missouri-Kansas City, Kansas City, MO 64110 USA; 28 Department of Internal Medicine, Taichung Veterans General Hospital, Taichung, 40705 Taiwan; 29 The Institute for Translational Genomics and Population Sciences, LABioMed and Department of Pediatrics at Harbor-UCLA Medical Center, Torrance, CA 90095 USA; 30 Cardiovascular Health Research Unit, Departments of Medicine, Epidemiology and Health Services, University of Washington, Seattle, 98195 WA USA; 31 Cardiovascular Health Research Unit, Kaiser Permanente Washington Health Research Institute, 98101 Seattle, WA USA; 32 Center for Public Health Genomics, University of Virginia School of Medicine, Charlottesville, VA 22908 USA; 33 Division of Cardiology, Johns Hopkins University School of Medicine, Baltimore, MD 21205 USA; 34 Center for Non-Communicable Diseases, Karachi, 74800 Pakistan; 35 Department of Biostatistics and Epidemiology, Perelman School of Medicine, University of Pennsylvania, Philadelphia, PA 19104 USA

## Abstract

Less than 3% of protein-coding genetic variants are predicted to result in loss of protein function through the introduction of a stop codon, frameshift, or the disruption of an essential splice site; however, such predicted loss-of-function (pLOF) variants provide insight into effector transcript and direction of biological effect. In >400,000 UK Biobank participants, we conduct association analyses of 3759 pLOF variants with six metabolic traits, six cardiometabolic diseases, and twelve additional diseases. We identified 18 new low-frequency or rare (allele frequency < 5%) pLOF variant-phenotype associations. pLOF variants in the gene *GPR151* protect against obesity and type 2 diabetes, in the gene *IL33* against asthma and allergic disease, and in the gene *IFIH1* against hypothyroidism. In the gene *PDE3B*, pLOF variants associate with elevated height, improved body fat distribution and protection from coronary artery disease. Our findings prioritize genes for which pharmacologic mimics of pLOF variants may lower risk for disease.

## Abstract (web summary)

Examination of predicted loss-of-function (pLOF) genetic variants allows direct identification of genes with therapeutic potential. Here, Emdin et al. perform association analysis for 3759 pLOF variants with 24 traits and highlight protective variants against cardiometabolic and immune phenotypes.

## Introduction

A focused investigation of predicted loss-of-function (pLOF) variants provides several advantages when compared with analysis of other types of variants. First, analysis of pLOF variants may allow for the direct identification of a gene rather than a locus containing many candidate genes^1^. Second, pLOF variants provide directionality of effect, unlike non-coding regulatory variants which may increase or decrease expression of a given gene. Third, identification of pLOF variants which protect against disease may aid with prioritization of therapeutic target genes (e.g., the recent development of inhibitors of PCSK9 or ANGPTL3 which mimic human pLOF mutations protective against cardiovascular disease)^2–6^.

Here, we analyse pLOF variants in UK Biobank and other datasets to identify genes for which pharmacologic inhibition may protect against disease. We identify associations of pLOF variants with cardiometabolic and immune disease, prioritizing the genes *GPR151, IL33, GSDMB, IFIH1,* and *PDE3B* as potential therapeutic targets.

## Results

### Analysis of loss-of-function variants

In 405,569 individuals in UK Biobank (335,660 individuals of European ancestry and 69,909 individuals of Non-European ancestry, Supplementary Table 1), we analyzed the association of 3759 pLOF variants with six metabolic traits [body mass index (BMI), waist-to-hip ratio adjusted for body mass index (WHRadjBMI), height, systolic blood pressure (SBP), diastolic blood pressure (DBP), forced expiratory volume to forced vital capacity ratio (FEV1/FVC)], six cardiometabolic diseases (coronary artery disease, type 2 diabetes, atrial fibrillation, stroke, heart failure, venous thromboembolism) and twelve diseases with more than 5000 cases (allergic rhinitis, asthma, anxiety, breast cancer, cataract, cholelithiasis, depression, hypothyroidism, gastric reflux, osteoporosis, osteoarthritis, and psoriasis). The Variant Effect Predictor and associated LOFTEE plugin^7,8^ algorithms were used to annotate variants which were pLOF (1) nonsense mutations that resulted in early termination of a protein; (2) frameshift mutations due to insertions or deletions of DNA; or (3) splice-site mutations which result in an incorrectly spliced protein. For coronary artery disease, we pooled UK Biobank estimates with estimates from the CARDIoGRAM Exome consortium^9^. For height, we pooled UK Biobank estimates with estimates from the GIANT Exome Consortium^10^. Variants with a *P* < 5.5 × 10^−7^ [0.05/(24 outcomes × 3759 variants) in the combined analysis were considered significant. Quantile–quantile analysis was used to examine for the presence of population stratification. No evidence of inflation was observed (inflation factors <1.1; Supplementary Fig. 1).

We identified 18 new low-frequency or rare (<5%) pLOF variants associated with traits and diseases in UK Biobank (Table 1 and Supplementary Table 2). We also discovered 26 new common frequency (≥5%) pLOF variants associated with UK Biobank phenotypes (Supplementary Table 3). Variants identified within the same locus in prior genome-wide association studies (+500 Kb; 1 Mb total) for the same phenotype are provided (Supplementary Tables 4 and 5).

**Table 1:** Predicted loss-of-function variants with minor allele frequency <5% which are significantly associated with traits or diseases in UK Biobank

Outcome	Gene	pLOF variant	Location	EA	RA	AA change	Freq (%)	Beta	SE	*P*-value	Novel?	MHC locus?
Asthma	FLG	rs61816761	1:152285861	A	G	p.Arg501Ter	1.51	0.21	0.03	1.51 × 10^−15^	Yes	No
Asthma	HLA-DQB1	rs28688207	6:32628660	C	T	Splice Acceptor c.773−1A > G	3.14	−0.17	0.02	3.11 × 10^−15^	Yes	Yes
Asthma	IL33	rs146597587	9:6255967	C	G	Splice Acceptor c.487−1G > C	0.44	−0.54	0.06	7.79 × 10^−17^	No^15^	No
BMI	GPR151	rs114285050	5:145895394	A	G	p.Arg95Ter	0.78	−0.07	0.01	4.89 × 10^−8^	Yes	No
BMI	PKHD1L1	rs533623778	8:110523131	T	C	p.Arg769Ter	1.0 × 10^−4^	5.30	0.99	9.45 × 10^−8^	Yes	No
DBP	ENPEP	rs33966350	4:111431444	A	G	p.Trp413Ter	1.19	0.06	0.01	8.12 × 10^−10^	No^39^	No
DBP	BTN3A2	rs58367598	6:26370833	G	T	Splice Donor c.715 + 2T > G	3.75	0.03	0.01	2.03 × 10^−8^	Yes	Yes
DBP	TMC3	rs150843673	15:81624929	T	G	p.Ser1045Ter	2.14	0.05	0.01	8.16 × 10^−9^	Yes	No
Height	PDE11A	rs76308115	2:178879181	A	G	p.Arg57Ter	0.52	0.07	0.01	6.20 × 10^−11^	Yes	No
Height	CLHC1	rs114931154	2:55407644	T	A	Splice Donor c.1384 + 2T > A	1.26	−0.05	0.01	1.54 × 10^−11^	Yes	No
Height	CCDC66	rs150364083	3:56628033	T	C	p.Arg427Ter	0.58	0.05	0.01	2.09 × 10^−7^	Yes	No
Height	DAP	rs201354802	5:10761153	A	C	p.Glu10Ter	0.24	0.13	0.02	1.68 × 10^−8^	Yes	No
Height	TRIM40	rs115651142	6:30115320	T	G	Splice Donor c.602 + 1G > T	0.63	−0.08	0.01	1.16 × 10^−9^	Yes	Yes
Height	MICA	rs181430930	6:31378575	A	G	Splice Donor c.286 + 1G > A	0.26	−0.12	0.02	7.87 × 10^−8^	Yes	Yes
Height	PDE3B	rs150090666	11:14865399	T	C	p.Arg783Ter	0.06	0.24	0.04	9.32 × 10^−9^	Yes	No
Height	APOLD1	rs202116412	12:12879031	A	G	Splice Donor c.96 + 1G > A	0.03	0.12	0.02	3.06 × 10^−8^	Yes	No
Hypothyroidism	IFIH1	rs35337543	2:163136505	G	C	Splice Donor c.1641 + 1G > C	1.45	−0.27	0.04	2.95 × 10^−9^	Yes	No
Psoriasis	ZKSCAN3	rs73387810	6:28318166	A	G	Splice Donor c.−63 + 1G > A	0.86	0.55	0.08	4.18 × 10^−11^	Yes	Yes
Psoriasis	EGFL8	rs141826798	6:32134395	G	C	p.Arg74Ter	0.53	0.90	0.08	2.19 × 10^−26^	Yes	Yes
SBP	ENPEP	rs33966350	4:111431444	A	G	p.Trp413Ter	1.19	0.06	0.01	3.46 × 10^−9^	No^39^	No
SBP	GEM	rs138582164	8:95264265	A	G	p.Arg199Ter	0.04	0.30	0.06	1.93 × 10^−7^	No^40^	No
WHRadjBMI	PYGM	rs116987552	11:64527223	A	G	p.Arg50Ter	0.39	0.09	0.02	1.32 × 10^−7^	Yes	No

Beta in terms of standard deviations and reported for the effect allele

*pLOF* predicted loss-of-function, *EA* effect allele, *RA* reference allele, *AA Change* amino acid change, *Freq(%)* Frequency(%); *BMI* body mass index, *DBP* diastolic blood pressure, *SBP* systolic blood pressure, *WHRadjBMI* waist-to-hip ratio adjusted for body mass index

A locus-wide conditional analysis (±500 kb of the pLOF variant) was performed to determine the extent to which the identified pLOF variant signal was independent of other genetic variation at the locus. Independence of pLOF variants may provide increased confidence for a causal association. Independent variants at the loci of rare and low frequency pLOFs are reported in Supplementary Data 1 and independent variants at the loci of common frequency pLOFs are reported in Supplementary Data 2. Of the 16 low frequency pLOF variants outside of the MHC locus, 14 were identified as independent variants in the locus-wide conditional analysis. These include the *IL33* variant rs146597587, the *GPR151* variant rs114285050, the *IFIH1* variant rs35337543, and the *PDE3B* variant rs15009066 which are further analyzed in the text of this manuscript. Below, we highlight several of these associations.

### *GPR151*, obesity, and type 2 diabetes

**Fig. 1:**
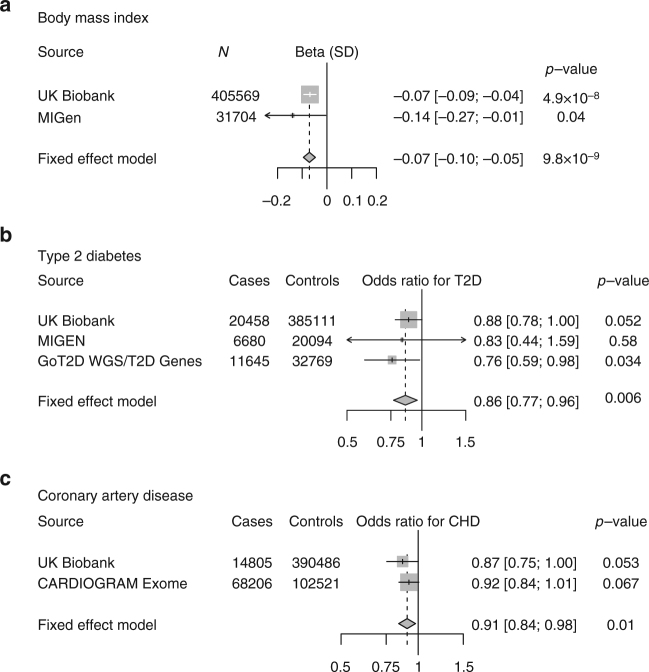
Association of a loss-of-function variant (p.Arg95Ter) in *GPR151* with **a** body mass index, **b** type 2 diabetes, and **c** coronary artery disease. Estimates were derived in UK Biobank using logistic regression, adjusted for age, sex, ten principal components of ancestry, and array type

In *GPR151* (encoding G-protein coupled receptor 151), the p.Arg95Ter variant (rs114285050, allele frequency 0.8% in European ancestry) was associated with reduced BMI (beta −0.07 standard deviations, −0.36 kg/m^2^, *P* = 4.9 × 10^−8^). We replicated this association in an independent cohort, the Myocardial Infarction Genetics Consortium (MIGen), where p.Arg95Ter carriers had reduced BMI (beta −0.14, *P* = 0.04; pooled beta −0.07, *P* = 9.8 × 10^−9^; Fig. 1). UK Biobank participants who carry one copy of p.Arg95Ter were at 12% lower odds of clinical obesity (BMI > 30 kg/m^2^). As obesity is a causal risk factor for type 2 diabetes and coronary artery disease, we examined whether p.Arg95Ter may provide protection against both diseases. p.Arg95Ter was associated with 14% lower odds of type 2 diabetes (OR 0.86; *P* = 0.006) and 9% lower odds of coronary artery disease (OR 0.91; *P* = 0.01; Fig. 1). Although *GPR151* encodes a G-protein coupled receptor of unknown function whose expression is limited to the central nervous system, recent studies tracing the lineage of neurons expressing *GRP151* have localized connections to hypothalamic neurons, a region of the brain important in the control of appetite^11^.

### *IL33, GSDMB*, and asthma

We identified several pLOF variants that associated with lower risk of asthma (Table 1). At *GSDMB* encoding gasdermin B, splice acceptor variant c.662-2A > G (rs11078928, allele frequency 46% in European ancestry) protected against asthma (OR 0.90 CI 0.89, 0.91, *P* = 6.7 × 10^−50^; Supplementary Table 3). This variant is in tight linkage disequilibrium (*r*^2^ = 0.99) with a previously identified non-coding variant in the *GSDMB* locus (rs2305480) associated with lower risk of asthma (*P* = 9.6 × 10^−8^)^12^. *GSDMB* c.662-2A > G is associated with lower expression of *GSDMB* transcripts^13^. Furthermore, overexpression of *GSDMB* causes airway remodeling and asthma symptoms in a mouse model^14^, suggesting that loss of GSDMB function may protect against asthma.

**Fig. 2:**
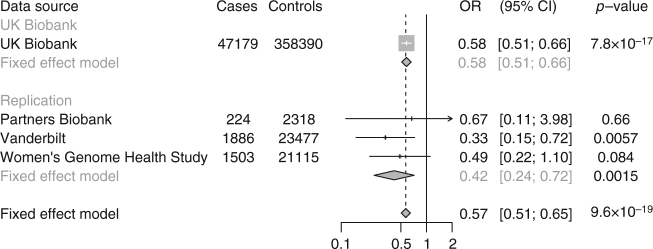
Association of *IL33* c.487-1G > C with asthma in UK Biobank, Partners Biobank, Vanderbilt eMERGE network and Women’s Genome Health Study. UK Biobank estimates were derived using logistic regression, adjusted for age, sex, ten principal components of ancestry, and array type. Partners Biobank and Vanderbilt estimates were derived using logistic regression, adjusted for age, sex, and principal components of ancestry. Women’s Genome Health Study estimates were derived using logistic regression, adjusted for age and principal components of ancestry

At the *IL33* gene, a splice acceptor site variant c.487-1G > C (rs146597587, allele frequency 0.004 in European ancestry) was observed to protect against asthma (OR 0.58 CI 0.51, 0.66, *P* = 7.8 × 10^−17^). This variant was recently identified as associated with lower blood eosinophil concentration at genome-wide significance and with lower risk of asthma at more modest levels of significance (*P* = 1.8 × 10^−4^)^15^. To further replicate the association of *IL33* c.487-1G > C with asthma, we examined the association of *IL33* c.487-1G > C with asthma in individuals from three additional studies (Partners Biobank, the Vanderbilt eMERGE network, and the Women’s Genome Health Study). *IL33* c.487-1G > C was associated with a protective effect of asthma in each data set. Overall, *IL33* c.487-1G > C was associated with 43% lower odds of asthma (OR 0.57 CI 0.51, 0.65, *P* = 9.6 × 10^−19^, Fig. 2), suggesting that IL33 inhibition may be a useful approach for treatment of asthma. Of note, an inhibitor of IL33 is currently under development for treatment of asthma^16^.

**Fig. 3:**
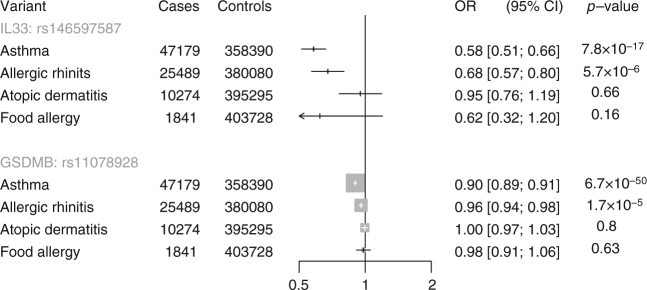
Association of predicted loss-of-function variant in *GSDMB* and *IL33* with allergic disease in UK Biobank. Estimates were derived in UK Biobank using logistic regression, adjusted for age, sex, ten principal components of ancestry, and array type

Asthma is often associated with other allergic phenotypes—atopic dermatitis, food allergy, and allergic rhinitis^17^. We therefore examined whether asthma-associated pLOF variants in *GSDMB* and *IL33* associate with a lower risk of other atopic disorders in UK Biobank. Both pLOF variants also protected against allergic rhinitis (Fig. 3). In contrast, nominal associations with atopic dermatitis and food allergy were not observed, although point estimates for food allergy were similar to asthma.

### *IFIH1* and autoimmune disorders

**Fig. 4:**
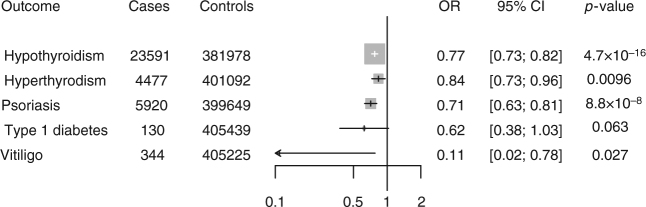
Association of predicted loss-of-function variants in *IFIH1* with thyroid disorders, type 1 diabetes, psoriasis, and vitiligo in UK Biobank. Estimates were derived in UK Biobank using logistic regression, adjusted for age, sex, ten principal components of ancestry, and array type

A splice donor variant in *IFIH1* (interferon induced with helicase C domain 1), c.1641 + 1G > C (rs35337543, allele frequency 1.5% in European ancestry), is associated with a reduced risk of hypothyroidism in UK Biobank participants (OR 0.77 CI 0.70, 0.85; *P* = 5 × 10^−9^; Table 1). A gene-based test combining four additional pLOF variants in *IFIH1* (rs35732034, rs201026962, rs35744605, rs148590996) similarly demonstrated protection against hypothyroidism in UK Biobank (OR 0.79 CI 0.72, 0.86; *P* = 4.4 × 10^−8^). Carriers of pLOF variants in *IFIH1* were also protected against hyperthyroidism (OR 0.84 CI 0.73, 0.96; *P* = 0.01; Fig. 4).

Common variants in the *IFIH1* locus have previously been associated with psoriasis^18^ and vitiligo^19^, while rare pLOF variants in *IFIH1* have been associated with a reduced risk of type 1 diabetes^20^. We therefore examined whether carriers of pLOF variants in *IFIH1* in UK Biobank were protected against these diseases. Carriers of pLOF variants in *IFIH1* were protected against type 1 diabetes, psoriasis, and vitiligo in UK Biobank (Fig. 4). These results suggest that *IF1H1* pLOF variants alter risk for a range of autoimmune diseases.

In addition, an exploratory analysis demonstrated a nominally lower risk of coronary artery disease among *IFIH1* pLOF carriers. Pooling UK Biobank and MIGen, *IFIH1* pLOF carriers were protected against coronary artery disease (OR 0.92 CI 0.87, 0.98; *P* = 0.009). To examine whether this may be a chance finding, we examined whether the common *IFIH1* missense variant rs1990760, previously identified as associated with autoimmune disorders, also associated with risk of coronary artery disease. The T allele of rs1990760 (frequency 41%) associated with a reduced risk of hypothyroidism (OR 0.92 CI 0.90, 0.94; *P* = 9.3 × 10^−17^) in UK Biobank. Pooling UK Biobank and CARDIOGRAM Exome, the T allele of rs1990760 also associated with a lower risk of coronary artery disease (OR 0.97 CI 0.96, 0.99; *P* = 2.5 × 10^−5^), providing complementary evidence that *IFIH1* may influence coronary artery disease risk.

### *PDE3B* and body fat distribution

**Fig. 5:**
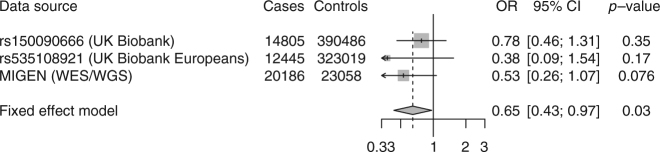
Association of predicted loss-of-function variants in *PDE3B* with coronary artery disease. Estimates were derived in UK Biobank using logistic regression, adjusted for age, sex, ten principal components of ancestry, and array type. Estimates were derived in MIGEN (Myocardial Infarction Genetics Consortium) using logistic regression, adjusted for sex and five principal components of ancestry

At *PDE3B* encoding the gene phosphodiesterase 3B, p.Arg783Ter (rs150090666, allele frequency 0.0006 in European ancestry) associated with elevated height (beta 0.24, *P* = 9.3 × 10^−9^). Targeted deletion of *Pde3b* in mice leads to white adipose tissue gaining characteristics of brown adipose tissue^21^, a reduction in adipocyte size^22^, smaller fat deposits^23^ and reduced atherosclerosis^24^. We therefore studied the association of *PDE3B* p.Arg783Ter with metabolic phenotypes in UK Biobank and/or MIGen, where 36,581 individuals have been sequenced for the 16 exons of the *PDE3B* gene. In UK Biobank, which lacks direct measurements of blood lipids, carriers of p.Arg783Ter carriers were at reduced risk of physician-diagnosis of hypercholesterolemia (OR 0.52, *P* = 0.0002). Pooling UK Biobank and MIGen, pLOF carriers in *PDE3B* had reduced WHRadjBMI (beta −0.15, *P* = 0.0005). As genetic predisposition to improved body fat distribution has been associated with a lower risk of coronary artery disease^25^, we examined whether loss of *PDE3B* function protects against coronary artery disease. We aggregated rare *PDE3B* pLOFs in cases and compared this count with that controls. Across 14,805 cases in UK Biobank, the carrier frequency of pLOF in cases was 0.1% and in controls 0.2%. Across 20,186 cases in MIGen, the carrier frequency of pLOF was 0.05% and 0.1% in controls. Collectively, carrier status for *PDE3B* pLOFs was associated with reduced risk for coronary artery disease (OR 0.65 CI 0.43, 0.97; *P* = 0.03; Fig. 5).

Presence of homozygote individuals for pLOF variants in target genes may provide an in vivo demonstration of safety of pharmacologic inhibition of target genes. We therefore examined whether homozygotes for these pLoF variants were present in UK Biobank and in the gnomAD database^26^. At least one individual homozygous for a pLOF variant was identified in UK Biobank or the gnomAD database for the genes *GPR151*, *GSDMB*, *IL33* and *IFIH1*, and *PDE3B* (Supplementary Table 6).

## Discussion

In this study, we identified pLOF variants that protect against obesity (*GPR151*), asthma (*GSDMB*, *IL33*), autoimmune disorders (*IFIH1*), and coronary artery disease (*PDE3B*), prioritizing genes and pathways for which pharmacologic attempts to mimic these protective mutations might ameliorate disease.

Identification of protective loss-of-function variants has led to the development of therapeutics. Most notably, the discovery of missense and loss-of-function variants in *PCSK9* that lower LDL cholesterol and protect against coronary artery disease suggested that inhibition of PCSK9 may be a useful therapeutic strategy for prevention and treatment of cardiovascular disease^3,27^. These genetic studies were validated by a large-scale randomized trial demonstrating that a monoclonal antibody directed against *PCSK9* reduced the risk of recurrent cardiovascular events^4^. More recently, the discovery of loss-of-function variants in *ANGPTL3* that lower blood triglyceride levels and protect against coronary artery disease has suggested that ANGPTL3 inhibition may reduce blood triglyceride levels and risk of coronary artery disease^28,29^. ANGTPL3 inhibitors are in clinical development and have now been demonstrated to reduce blood triglyceride levels^5,6^.

Our findings identify putative therapeutic targets that, similar to PCSK9 and ANGPTL3, may be useful for prevention of disease. For obesity and type 2 diabetes, these results hightlight GPR151 as a potential therapeutic target. *GPR151* encodes a largely uncharacterized G protein-coupled receptor. The mechanism by which it influences risk of obesity and type 2 diabetes is unclear. However, it is highly expressed in the hypothalamus, a region of the brain known to be involved in appetite regulation^11^. Indeed, genetic variation in *MC4R*, which encodes the melanocortin 4 receptor, strongly influences obesity risk at a population level^30^. Similar to *GPR151*, *MC4R*is highly expressed in the hypothalamus and is involved in appetite regulation^31^.

PDE3B inhibition may be a useful therapeutic strategy to improve body fat distribution and reduce risk of coronary artery disease. Unlike *GPR151*, for which no pharmacologic inhibitor is currently in clinical use, an inhibitor of *PDE3B* is available. Cilostazol is a non-selective pharmacologic inhibitor of both phosphodiesterase 3B and the related isoform phosphodiesterase 3A^32^. In a small randomized trial including 211 participant, cilostazol significantly reduced restenosis after percutaneous coronary balloon angioplasty^33^. The association of *PDE3B* pLOFs with improved body fat distribution, reduced risk of hypercholesterolemia and reduced risk of coronary artery disease suggests that selective inhibition of PDE3B may be useful for multiple features of metabolic syndrome.

We identified pLOF variants in *IL33* (encoding interleukin 33) and *GSDMB* (encoding gasdermin B) as associated with a lower risk of asthma. The *IL33* variant rs146597587 was recently found to be associated with lower blood eosinophil concentration at genome-wide significance and with lower risk of asthma at more modest levels of significance (*P* = 1.8 × 10^−4^)^15^. Consistent with these findings, induction of antibodies against IL33 by vaccination induces protection against airway inflammation in a mouse model of asthma^34^. An inhibitor of IL33 is currently under development for treatment of asthma^16^. In contrast to IL33, no inhibitor for GSDMB is in clinical development. These findings suggest that IL33 and GSDMB inhibition may both be useful therapeutic strategies for treatment of asthma and allergic disease.

Although our restriction of the present analysis to pLOF variants increases the likelihood of identifying causal variants substantially, it remains possible that a highly correlated nearby variant could be driving the association in some cases. Future functional studies may permit additional validation of causal variants.

In summary, we associated pLOF variants with a range of biomarker and disease phenotypes in a large, national biobank and identified several new genes in which pLOF variants protect against disease, prioritizing these genes for therapeutic targeting. More generally, large-scale analysis of pLOF variants is emerging as a useful tool for therapeutic target identification and validation.

## Methods

### Gene and variant annotation

Variants in hg19 coordinates were annotated with information from Ensembl release 82 using Variant Effect Predictor (VEP)^7^. Only pLOFs, defined as premature stop (nonsense), canonical splice-sites (splice-donor or splice-acceptor) or insertion/deletion variants that shifted frame (frameshift) were annotated as predicted loss-of-function (pLOF), using the “--pick-allele” annotation. PLOFs as defined by VEP were then merged with publicly available data from the Exome Aggregation Consortium (ExAC), Version 0.3.1, to confirm consistency in variant annotation^26^.

We identified 3759 pLOF variants in UK Biobank with an info score greater than 0.3 (Supplementary Table 1). We used a Bonferroni corrected *P* value of 5.5 × 10^−7^ to denote significance [0.05/(3759 variants × 24 outcomes) = 5.5 × 10^−7^] in our primary pLOF analysis.

### Study design

We analyzed the association of pLOF variants with 24 phenotypes: cardiovascular, metabolic and pulmonary phenotypes: six metabolic traits (body mass index, waist-to-hip ratio, height, systolic blood pressure, diastolic blood pressure, and forced expiratory volume to forced vital capacity ratio), six cardiometabolic diseases (coronary artery disease, type 2 diabetes, atrial fibrillation, stroke, heart failure, and venous thromboembolism) and 12 diseases with more than 5000 cases (allergic rhinitis, asthma, anxiety, breast cancer, cataract, cholethiasis, depression, hypothyroidism, gastric reflux, osteoporosis, osteoarthritis, and psoriasis; Supplementary Table 4). All six metabolic traits were inverse normalized prior to analysis, with adjustment for age and sex. Forced expiratory volume to forced vital capacity ratio was additionally adjusted for height. To adjust for the presence of antihypertensive medication, we added 15 mm Hg to systolic blood pressure and 10 mm Hg to diastolic blood pressure of individuals on antihypertensive medication at baseline, as in the International Consortium for Blood Pressure genome-wide association study^35^. Definitions for disease outcomes in UK Biobank are provided (Supplementary Table 7).

In UK Biobank, analysis was performed separately in unrelated individuals of European and Non-European ancestry. Estimates for variants were then pooled using inverse-variance weighted-fixed effects meta-analysis. For coronary artery disease, estimates for variants from UK Biobank were additionally pooled with the effect of variants in the CARDIOGRAM Exome consortium using inverse variance weighted-fixed effects meta-analysis^9^. For height, estimates of variants in UK Biobank were pooled with the GIANT Height Exome consortium using inverse variance weighted-fixed effects meta-analysis^10^.

### Genotyping and quality control

Phasing and imputation were performed centrally, by UK Biobank, using a reference panel combining UK10k and 1000 Genomes samples. 39,235,157 variants included in the Haplotype Reference Consortium were imputed. As recommended by UK Biobank, we excluded any samples with an imputation quality <0.3 as well as pLOF variants which were not included in the Haplotype Reference Consortium. Mitochondrial genetic data and sex chromosomes were excluded from this analysis. Individual level genetic data was available from individuals in UK Biobank, after excluding one related individual of each related pair of individuals, individuals whose genetic sex did not match self-reported sex and individuals with an excess of missing genotype calls or more heterozygosity than expected.

We analysed 3759 variants identified as pLOF variants in UK Biobank. PLINK 2 software was used to examine the association of these variants with traits and disease in UK Biobank under the assumption of additive effects (https://www.cog-genomics.org/plink/2.0/). Adjustment was performed for age, sex, ten principal components of ancestry, and array type.

### Conditional analysis

A locus-wide conditional analysis (±500 kb of the pLOF variant) was performed to determine the extent to which the identified pLOF variant signal was independent of other genetic variation at the locus. We iteratively performed association analyses conditional on the top variants at each locus, until no variants were below the Bonferroni-adjusted threshold for significance (*P* < 5.5 × 10^−7^). A statistically significant and independent signal for the pLOF variant provides increased confidence for a causal association.

### Analysis of PDE3B association with coronary artery disease

We aimed to analyse the association of pLOF variants in *PDE3B* with coronary artery disease in a combined analysis of UK Biobank and the Myocardial Infarction Genetics Consortium (MIGen). Replication was performed in MIGen rather than the CARDIOGRAM Exome consortium as rs150090666 was not included in the exome chip analysis of the CARDIOGRAM Exome consortium^9^. Estimates of the association of rs150090666 with coronary artery disease in UK Biobank were derived as described above, using logistic regression with adjustment for age, sex, ten principal components of ancestry, and a dummy variable for array type. An additional pLOF variant, rs535108921, present in UK Biobank, was also analysed for association with coronary artery disease, as above.

pLOFs variants in *PDE3B* were identified in the MIGen Consortium using exome sequencing or whole genome sequencing, as previously described^36–38^. Studies included in the MIGen consortium were: (1) the Italian Atherosclerosis Thrombosis and Vascular Biology (ATVB) study (dbGaP Study Accession phs000814.v1.p1); (2) the Exome Sequencing Project Early-Onset Myocardial Infarction (ESP-EOMI) study(9); (3) a nested case-control cohort from the Jackson Heart Study (JHS); (4) the South German Myocardial Infarction study (dbGaP Study Accession phs000916.v1.p1); (5) the Ottawa Heart Study (OHS) (dbGaP Study Accession phs000806.v1.p1); (6) the Precocious Coronary Artery Disease (PROCARDIS) study (dbGaP Study Accession phs000883.v1.p1); (7) the Pakistan Risk of Myocardial Infarction Study (PROMIS) (dbGaP Study Accession phs000917.v1.p1); (8) the Registre Gironi del COR (Gerona Heart Registry or REGICOR) study (dbGaP Study Accession phs000902.v1.p1); (9) the Leicester Myocardial Infarction study (dbGaP Study Accession phs001000.v1.p1); (10) the BioImage study (dbGaP Study Accession phs001058.v1.p1); (11) the North German Myocardial Infarction study (dbGaP Study Accession phs000990.v1.p1); (12) Multi-Ethnic Study of Atherosclerosis (dbGaP Study Accession: phs000209.v2.p1); (13) Variation In Recovery: Role of Gender on Outcomes of Young AMI cohort; and (14) Taiwan Metabochip Consortium.

The Burrows–Wheeler Aligner algorithm was used to align reads from participants to the reference genome (hg19). The GATK HaploTypeCaller was used to jointly call variants. Metrics including Variant Quality Score Recalibration (VQSR), quality over depth, and strand bias were then used to filter variants. We excluded samples which were related to other samples, which had high ratios of heterozygous to non-reference homozygous genotypes, which had high missing genotypes, which had a discordant genetic gender relative to reports gender, and samples which were discordant relative to genotype data.

After variant calling and quality control, the Variant Effect Predictor^7,8^ was used to annotate variants which were pLOF: (1) nonsense mutations that resulted in early termination of *PDE3B* (2) frameshift mutations due to insertions or deletions of DNA; or (3) splice-site mutations which result in an incorrectly spliced protein (Supplementary Table 8). For analysis of rare pLOF variants, we pooled rare pLOF variants in MIGen, testing for the association of a pLOF with coronary artery disease using logistic regression, after adjustment for age, sex, cohort, and five principle components of ancestry. We meta-analysed the association of pLOFs with coronary artery disease in MIGen combined with UK Biobank.

### Replication of IL33 finding

To replicate the association of rs146597587, a splice site variant in *IL33*, with asthma, we pooled estimates of the association of rs146597587 with asthma from Partners Biobank, from the Vanderbilt eMERGE network and from the Women’s Genome Health Study. In Partners Biobank, rs146597587 was imputed (info score of 0.77) in 2542 individuals. The association of rs146597587 with asthma (hospitalization for ICD9 code 493) was estimated using logistic regression, adjusted for age, sex, and five principal components of ancestry. In the Vanderbilt eMERGE network, rs146597587 was genotyped in 25,363 individuals using the Illumina Exome BeadChip. The association of rs146597587 with asthma (hospitalization for ICD9 code 493) was estimated using logistic regression, adjusted for age, sex, and principal components of ancestry. In Women’s Genome Health Study, rs14659758 was genotyped in 22,618 individuals using the Illumina Exome. The association of rs14659758 with asthma (hospitalization for ICD9 code 493 or ICD10 code J45) was estimated using logistic regression, adjusted for age and principal components of ancestry.

### Data availability

All individual-level data from UK Biobank can be accessed by applying to the UK Biobank Central Access Committee (http://www.ukbiobank.ac.uk/register-apply/).

## Electronic supplementary material

Supplementary Information

Description of Additional Supplementary Files

Supplementary Data 1

Supplementary Data 2
