# Supplementary material for: Analysis of predicted loss-of-function variants in UK Biobank identifies variants protective for disease
Source: Nat Commun. 2018 Apr 24;9:1613. doi: 10.1038/s41467-018-03911-8 (PMC5915445; doi:10.1038/s41467-018-03911-8)
Supplement: Supplementary file 1 — Supplementary Information [file 41467_2018_3911_MOESM1_ESM.pdf]

Supplementary Information

Emdin et al. Analysis of predicted loss of function variants in UK Biobank identifies variants protective for disease.

Supplementary Table 1. Characteristics of individuals in UK Biobank.

	European Ancestry	Non-European Ancestry
N Individuals	335660	69909
Age ± SD, years	57 ± 8	55 ± 8
Male, n (%)	155753 (46%)	31440 (45%)
UK BiLEVE Array, n (%)	43 528 (13%)	5097 (7%)
Systolic Blood Pressure ± SD, mm Hg*	143 ± 22	140 ± 22
Diastolic Blood Pressure ± SD, mm Hg*	84 ± 12	84 ± 12
Body Mass Index ± SD, kg/m2	27 ± 5	27 ± 5
Waist-to-Hip Ratio ± SD	0.87 ± 0.09	0.87 ± 0.09
Coronary Heart Disease, n (%)	12445 (3.7%)	2360 (3.3%)

Abbreviations: SD, standard deviation

Supplementary Table 2. Characteristics of genes identified in low frequency (<5%) predicted loss-of-function variant analysis.

Gene	Description of Gene
FLG	FLG encodes filaggrin, a protein which crosslinks keratin fibers to form a protein-lipid matrix in the epidermis which prevents water loss and infection. Loss of function variants in FLG have been identified as a cause of atopic dermatitis.[7]
HLA-DQB1	HLA-DQB1 encodes the beta 1 subunit of the class II major histocompatibility complex, present on antigen-presenting cells to display antigens. Polymorphisms in this gene are associated with a range of autoimmune disorders.[8]
IL33	IL33 encodes interleukin-33, a cytokine that is an inducer of T helper 2 cell responses. Injection of IL33 into mice induces of eosinophilia. Expression of IL33 is elevated among individuals with severe asthma.[9]
GPR151	*GPR151* encodes a G-protein coupled receptor of unknown function whose expression seems limited to the central nervous system. Of note, recent lineage tracing studies have localized connections to hypothalamic neurons, a region of the brain important in the control of appetite.[10]
PKHD1L1	PKHD1L1 encodes fibrocystin-L, a large (446 kilodalton protein) predicted to have a single transmembrane domain. Fiibrocystin-L is homologous to PKHD1, the autosomal recessive polycystic kidney disease gene, but is of unknown function.[11]
ENPEP	ENPEP encodes glutamyl aminopeptiase, an enzyme that degrades angiotensin II and promotes vasodilation.[12]
BTN3A2	BTN3A2 encodes a butyrophilin family member. Other butyrophilin family members regulate T cell activation [13] and variants in BTN3A2 have been reported to be associated with gastric cancer[14] and hepatitis C infection. [15] However, the function of BTN3A2 is unclear.[15]
TMC3	TMC3 encodes transmembrane channel-like 3, a protein homologous to a known ion channel but of unclear function.[16]
PDE11A	PDE11A encodes phosphodiesterase 11A which hydrolyzes both cAMP and cGMP and is expressed in skeletal muscle, prostate, kidney, liver, pituitary, and salivary glands and testis.[17] Loss of function variants in phosphodiesterase 11A have been identified as a cause of adrenocortical hyperplasia.[18]
CLHC1	CLHC1 encodes clathrin heavy chain linker domain containing 1, a protein of unknown function.
CCDC66	CCDC66 encodes coiled-coil domain containing 66 is a widely expressed protein of uncertain function. Loss of function variants in coiled-coil domain containing 66 have been reported to cause retinal degeneration and dysfunction in mice.[19]
DAP	DAP encodes death-associated protein 1, a small cytosolic mediator of interferon-gamma induced cell death.[20] Reduced expression of death-associated protein 1 in tumor tissues has been associated with an increased risk of death in colorectal cancer patients[21] and in breast cancer patients.[22]
TRIM40	TRIM40 encodes tripartite motif containing 40, a member of the tripartite motif containing family which have been reported to have roles in ubiquitination. TRIM40 has been reported to prevent inflammation in the gastrointenstinal tract

	by inhibiting nuclear factor-kappaB.[23]
MICA	MICA encodes MHC class I polypeptide-related sequence A, a protein that is highly expressed on the surface of intestinal epithelial cells during stress.[24] By binding to receptors on T cells and natural killer cells, MICA promotes a cytolytic response, thus inducing an anti-tumor response when expressed on tumor cells.[24] MICA has also been reported to be expressed by intestinal epithelial during cytomegalovirus infection[25] and in the intestinal epithelial of active celiac disease patients.[26]
PDE3B	*PDE3B* encodes the gene phosphodiesterase 3B, an adipocyte-expressed enzyme that hydrolyzes cyclic adenosine monosphosphate (cAMP) and inhibits lipolysis in response to insulin binding to the insulin receptor. [27] *PDE3B* knockout mice have been reported to have reduced aortic atherosclerosis and markers of inflammation.[28] Furthermore, *PDE3B* knockout reduced infarct size in a mouse coronary artery ligation model of myocardial infarction.[29] Of note, cilostazol is an approved medicine that is a non-selective pharmacologic inhibitor of both phosphodiesterase 3B and the related isoform phosphodiesterase 3A.[29] In a small 211 participant randomized trial, cilostazol significantly reduced restenosis after percutaneous coronary balloon angioplasty.[30]
APOLD1	APOLD1 encodes apolipoprotein L domain containing 1, a protein of unclear function. Apolipoprotein L domain containing 1 is expressed in vascular endothelial cells and has been reported to been induced in the endothelium by electrical or chemical stimulation. [31]
IFIH1	IFIH1 encodes interferon induced with helicase C domain 1, a cytoplasmic receptor that induces interferon signaling upon binding to viral RNA. [32] Gain of function mutations in IFIH1 cause Aicardi-Goutières syndrome, a rare genetic disorder characterized by lymphocytosis in the cerebral spinal fluid and cerebral atropy. [32] Common variants in the *IFIH1* locus have previously been identified as associated with psoriasis[33] and vitiligo[34], while rare pLOF variants in *IFIH1* have been associated with a reduced risk of type 1 diabetes[35].
ZKSCAN3	ZKSCAN3 (zinc finger with KRAB and SCAN domains 3) encodes a zinc finger family DNA binding protein. ZKSCAN3 represses transcription of autophagy genes[36] and is upregulated in human colon cancer cells. [37]
EGFL8	EGFL8 encodes epidermal growth factor like 8, a gene of unknown function.
GEM	GEM encodes a GTP binding protein induced in T-cells in response to mitogen stimulation.[38] GEM has been reported to down regulate voltage gated calcium channel activity.[39] The physiologic function of GEM is unclear.
PYGM	PYGM encodes glycogen phosphorylase, an enzyme expressed in skeletal muscle that hydrolyzes glycogen into glucose-1-phosphate for energy during exercise.[40] Homozygous loss of function variants in PYGM cause McArdle disease, which is characterized by the presence of muscle pain and weakness upon exercise that is immediately relieved upon stopping and the absence of lactate formation during exercise.[41]

Supplementary Table 3. Common (≥5% frequency) loss of function variants significantly associated with traits and disease in UK Biobank.

Outcome	Gene	Variant	Location	EA	RA	Consequence	Frequency (%, European)	Beta	SE	P-value	Novel?	MHC Locus?
Asthma	GSDMB	rs11078928	17:38064469	C	T	Splice Acceptor (c.662-2A>G)	46	-0.10	0.007	$6.66*10^{-50}$	No[1]	No
Asthma	BTN3A2	rs71557335	6:26368279	A	G	Splice Donor (c.-6+1G>A)	13	0.06	0.010	$1.67*10^{-8}$	Yes	Yes
Asthma	CCHCR1	rs72856718	6:31125257	A	C	p.Glu41Ter	7	-0.13	0.015	$2.06*10^{-18}$	Yes	Yes
BMI	IQCK	rs4782272	16:19729016	C	G	Splice Acceptor (c.-612-1G>C)	18	-0.02	0.003	$1.12*10^{-10}$	Yes	No
BMI	ANKDD1B	rs34358	5:74965122	A	G	p.Trp480Ter	36	-0.03	0.002	$5.94*10^{-30}$	No[2]	No
CHD	MOB3C	rs6671527	1:47080679	A	G	p.Arg24Ter	47	-0.03	0.006	$2.31*10^{-7}$	Yes	No
CHD	LPL	rs328	8:19819724	G	C	p.Ser474Ter	10	-0.07	0.010	$1.62*10^{-11}$	No[3]	No
DBP	OR4X2	rs7120775	11:48266736	G	C	p.Tyr27Ter	15	-0.02	0.003	$1.49*10^{-12}$	Yes	No
DBP	OR4C11	rs75423534	11:55371381	A	G	p.Gln157Ter	11	-0.02	0.004	$2.69*10^{-9}$	Yes	No
DBP	CFL1	rs1939212	11:65626701	T	C	Splice Donor (c.-532+1G>A)	30	0.01	0.003	$9.09*10^{-8}$	Yes	No
DBP	FUT2	rs601338	19:49206674	A	G	p.Trp154Ter	50	-0.02	0.002	$6.05*10^{-11}$	Yes	No
DBP	BTN3A2	rs71557335	6:26368279	A	G	Splice Donor (c.-6+1G>A)	13	-0.02	0.003	$2.36*10^{-8}$	Yes	Yes
FEV1/FVC	BTN3A2	rs71557335	6:26368279	A	G	Splice Donor (c.-6+1G>A)	13	-0.02	0.003	$1.19*10^{-7}$	Yes	Yes
FEV1/FVC	ZSCAN9	rs76542212	6:28198122	T	C	p.Arg193Ter	7	0.02	0.005	$5.07*10^{-8}$	Yes	Yes
FEV1/FVC	CCHCR1	rs3130453	6:31124849	T	C	p.Trp78Ter	49	-0.02	0.002	$7.92*10^{-12}$	Yes	Yes
FEV1/FVC	CCHCR1	rs72856718	6:31125257	A	C	p.Glu41Ter	7	0.03	0.005	$4.32*10^{-9}$	Yes	Yes
Height	AKR1E2	rs12240276	10:4889403	T	C	p.Arg301Ter	11	-0.02	0.002	$6.19*10^{-15}$	Yes	No
Height	PATE4	rs11220236	11:125707761	C	A	Splice Acceptor (c.59-2A>C)	41	-0.01	0.002	$1.09*10^{-8}$	Yes	No
Height	OR4X2	rs7120775	11:48266736	G	C	p.Tyr27Ter	15	0.02	0.003	$1.08*10^{-8}$	No[4]	No
Height	DYX1C1	rs57809907	15:55722882	A	C	p.Glu417Ter	10	-0.02	0.003	$2.30*10^{-9}$	Yes	No
Height	TBC1D29	rs111780165	17:28887134	A	G	Splice Acceptor (c.13-1G>A)	7	-0.06	0.004	$3.47*10^{-40}$	No[4]	No
Height	SLC35G6	rs7214088	17:7386280	A	G	p.Trp326Ter	26	0.03	0.003	$3.49*10^{-37}$	No[4]	No
Height	ACYP2	rs1363061	2:54284441	A	G	p.Trp74Ter	11	-0.02	0.003	$4.28*10^{-9}$	Yes	No

Height	CPN2	rs4974539	3:194061907	A	G	p.Gln509Ter	38	-0.01	0.002	$1.88*10^{-8}$	Yes	No
Height	OR2J1	rs2394517	6:29069299	T	C	p.Gln194Ter	41	-0.03	0.002	$5.21*10^{-41}$	No[4]	Yes
Height	CCHCR1	rs72856718	6:31125257	A	C	p.Glu41Ter	7	-0.05	0.004	$4.38*10^{-36}$	No[4]	Yes
Hypothyroidism	BTN3A2	rs71557335	6:26368279	A	G	Splice Donor (c.-6+1G>A)	13	0.09	0.014	$4.88*10^{-10}$	Yes	Yes
Hypothyroidism	CCHCR1	rs72856718	6:31125257	A	C	p.Glu41Ter	7	0.14	0.018	$4.33*10^{-14}$	No[5]	Yes
Psoriasis	OR2J1	rs2394517	6:29069299	T	C	p.Gln194Ter	41	0.13	0.019	$2.46*10^{-12}$	No[6]	Yes
Psoriasis	CCHCR1	rs3130453	6:31124849	T	C	p.Trp78Ter	49	0.34	0.019	$1.54*10^{-71}$	No[6]	Yes
SBP	OR4X2	rs7120775	11:48266736	G	C	p.Tyr27Ter	15	-0.03	0.003	$9.37*10^{-16}$	Yes	No
SBP	OR4C11	rs75423534	11:55371381	A	G	p.Gln157Ter	11	-0.03	0.004	$3.04*10^{-10}$	Yes	No
SBP	CFL1	rs1939212	11:65626701	T	C	Splice Donor (c.-532+1G>A)	30	0.02	0.003	$8.32*10^{-10}$	Yes	No
SBP	TNK1	rs7220814	17:7290695	G	A	Splice Acceptor (c.1398-2A>G)	6	-0.02	0.005	$1.89*10^{-7}$	Yes	No
SBP	FUT2	rs601338	19:49206674	A	G	p.Trp154Ter	50	-0.02	0.002	$3.87*10^{-11}$	Yes	No
WHRadjBMI	SLC35G6	rs7214088	17:7386280	A	G	p.Trp326Ter	26	0.01	0.003	$4.01*10^{-7}$	Yes	No
WHRadjBMI	BTN3A2	rs71557335	6:26368279	A	G	Splice Donor (c.-6+1G>A)	13	-0.02	0.003	$4.06*10^{-13}$	Yes	Yes

Abbreviations: EA, effect allele; RA, reference allele; SE, standard error

[1]Variant identified as independent in sequential conditional analysis.

Supplementary Table 4. Variants previously reported in genome wide association studies within the same loci as identified low frequency loss of function variants.

Outcome	Gene	Rare Variant	Location	Frequency (%, European)	Previous Variant in Locus?	Variant	Location	Frequency (%)
Asthma	FLG	rs61816761	1:152285861	1.51	No			
Asthma	HLA-DQB1	rs28688207	6:32628660	3.14	Yes[2]	rs9273349	6:32733847	42
Asthma	IL33	rs146597587	9:6255967	0.44	Yes[2]	rs1342326	9:6180076	16
BMI	GPR151	rs114285050	5:145895394	0.78	No			
BMI	PKHD1L1	rs533623778	8:110523131	$1.0*10^{-4}$	No			
DBP	ENPEP	rs33966350	4:111431444	1.19	No			
DBP	BTN3A2	rs58367598	6:26370833	3.75	No			
DBP	TMC3	rs150843673	15:81624929	2.14	No			
Height	PDE11A	rs76308115	2:178879181	0.52	Yes[3]	rs3821008	2:178682329	12
Height	CLHC1	rs114931154	2:55407644	1.26	No			
Height	CCDC66	rs150364083	3:56628033	0.58	Yes[3]	rs9835332	3:56642722	46
Height	DAP	rs201354802	5:10761153	0.24	No			
Height	TRIM40	rs115651142	6:30115320	0.63	Yes[3]	rs9404952	6:29912144	44
Height	MICA	rs181430930	6:31378575	0.26	Yes[3]	rs6457374	6: 31380240	27
Height	PDE3B	rs150090666	11:14865399	0.06	No			
Height	APOLD1	rs202116412	12:12879031	0.03	Yes[3]	rs1420023	12: 12767378	12
Hypothyroidism	IFIH1	rs35337543	2:163136505	1.45	Yes[4,5]	rs1990760		
Psoriasis	ZKSCAN3	rs73387810	6:28318166	0.86	No			
Psoriasis	EGFL8	rs141826798	6:32134395	0.53	Yes[6]	rs4406273	6: 31266090	26
SBP	ENPEP	rs33966350	4:111431444	1.19	No			
SBP	GEM	rs138582164	8:95264265	0.04	No			
WHRadjBMI	PYGM	rs116987552	11:64527223	0.39	No			

Supplementary Table 5. Variants previously reported within the same loci as identified common loss of function variants.

Outcome	Gene	Variant	Location	Frequency (%, European)	Previous Variant in Locus?	Variant	Location	Frequency (%)
Asthma	GSDMB	rs11078928	17:38064469	46	Yes[2]	rs2305480	17:38062196	45
Asthma	BTN3A2	rs71557335	6:26368279	13	No			
Asthma	CCHCR1	rs72856718	6:31125257	7	No			
BMI	IQCK	rs4782272	16:19729016	18	Yes[7]	rs11641786	16:19844865	14
BMI	ANKDD1B	rs34358	5:74965122	36	Yes[7]	rs253414	5:74956517	34
CHD	MOB3C	rs6671527	1:47080679	47	No			
CHD	LPL	rs328	8:19819724	10	Yes[8]	rs264	8:19813180	14
DBP	OR4X2	rs7120775	11:48266736	15	No			
DBP	OR4C11	rs75423534	11:55371381	11	No			
DBP	CFL1	rs1939212	11:65626701	30	No			
DBP	FUT2	rs601338	19:49206674	50	No			
DBP	BTN3A2	rs71557335	6:26368279	13	No			
FEV1/FVC	BTN3A2	rs71557335	6:26368279	13	No			
FEV1/FVC	ZSCAN9	rs76542212	6:28198122	7	No			
FEV1/FVC	CCHCR1	rs3130453	6:31124849	49	Yes[9]	rs2857595	6:31568469	21
FEV1/FVC	CCHCR1	rs72856718	6:31125257	7	Yes[9]	rs2857595	6:31568469	21
Height	AKR1E2	rs12240276	10:4889403	11	Yes[10]	rs2281880	10: 104269217	46
Height	PATE4	rs11220236	11:125707761	41	No			
Height	OR4X2	rs7120775	11:48266736	15	Yes[3]	rs10838835	11: 48246457	12
Height	DYX1C1	rs57809907	15:55722882	10	No			
Height	TBC1D29	rs111780165	17:28887134	7	Yes[10]	rs3816780	17: 29161358	11
Height	SLC35G6	rs7214088	17:7386280	26	Yes[3]	rs11658168	17: 7406134	38
Height	ACYP2	rs1363061	2:54284441	11	Yes[3]	rs7588499	2:54728276	48
Height	CPN2	rs4974539	3:194061907	38	No			

Height	OR2J1	rs2394517	6:29069299	41	Yes[3]	rs3129109	6:29084232	37
Height	CCHCR1	rs72856718	6:31125257	7	Yes[3]	rs2073724	6:31125257	5
Hypothyroidism	BTN3A2	rs71557335	6:26368279	13	No			
Hypothyroidism	CCHCR1	rs72856718	6:31125257	7	Yes[11]	rs2517532	6:31126386	40
Psoriasis	OR2J1	rs2394517	6:29069299	41	No			
Psoriasis	CCHCR1	rs3130453	6:31124849	49	Yes[12]	rs4406273	6:31266090	9
SBP	OR4X2	rs7120775	11:48266736	15	No			
SBP	OR4C11	rs75423534	11:55371381	11	No			
SBP	CFL1	rs1939212	11:65626701	30	No			
SBP	TNK1	rs7220814	17:7290695	6	No			
SBP	FUT2	rs601338	19:49206674	50	No			
WHRadjBMI	SLC35G6	rs7214088	17:7386280	26	No			
WHRadjBMI	BTN3A2	rs71557335	6:26368279	13	No			

Supplementary Table 6. Frequency of loss of function homozygotes in UK Biobank and in gnoMAD.

		Number of Homozygotes	
Gene	Variant	UK Biobank	gnoMAD
GPR151	rs114285050	30 of 405569	13 of 138592
GSDMB	rs11078928	85535 of 405569	14909 of 90689
IL33	rs146597587	5 of 405569	1 of 138239
IFIH1	rs35732034	41 of 405569	7 of 131469
PDE3B	rs150090666	0 of 405569	1 of 138583

Supplementary Table 7. Definitions of disease outcomes in UK Biobank.

Outcome	Definition (UK Biobank unless otherwise specified)
Cardiometabolic disease	
Coronary heart disease	Inverse variance weighted fixed effects meta-analysis of CARDIOGRAM Exome Consortium[42] outcome (coronary heart disease) and UK Biobank outcome:<br>(1) Myocardial infarction (MI), coronary artery bypass grafting, or coronary artery angioplasty documented in medical history at time of enrollment by a trained nurse or<br>(2) Hospitalization for ICD-10 code for acute myocardial infarction (I21.0, I21.1, I21.2, I21.4, I21.9) or<br>(3) Hospitalization for OPCS-4 coded procedure: coronary artery bypass grafting (K40.1-40.4, K41.1-41.4, K45.1-45.5) or<br>(4) Hospitalization for OPCS-4 coded procedure: coronary angioplasty ± stenting (K49.1-49.2, K49.8-49.9, K50.2, K75.1-75.4, K75.8-75.9)
Type 2 Diabetes	History of diabetes unspecified, type 2 diabetes during verbal interview with trained nurse or hospitalization for or death due to ICD code E11
Atrial fibrillation	History of atrial fibrillation or flutter during verbal interview with trained nurse or hospitalization for or death due to ICD code I48
Stroke	History of stroke, adjudicated by UK Biobank centrally as report of stroke during verbal interview with trained nurse or hospitalization for or death due to ICD code I60-64 *(http://biobank.ctsu.ox.ac.uk/crystal/refer.cgi?id=462)*
Heart failure	(History of heart failure during verbal interview with trained nurse or hospitalization for or death due to ICD code I11.0, I13.0, I13.2, I125.5, I42, I50
Venous thromboembolism	History of venous thromboembolism, deep vein thrombosis or pulmonary embolism during verbal interview with trained nurse or hospitalization for death due to I26, I80.1, I80.2, I81, or I82.0
Other diseases (>5000 cases)	
Allergic Rhinitis	History of allergic rhinitis/hayfever during verbal interview with trained nurse or hospitalization for or death due to ICD code J30
Asthma	History of asthma during verbal interview with trained nurse
Anxiety	(History of anxiety/panic attacks during verbal interview with trained nurse or hospitalization for or death due to ICD code F41
Breast cancer	History of breast cancer during verbal interview with trained nurse or hospitalization for or death due to ICD code C50
Cataract	History of cataract during verbal interview with trained nurse or hospitalization for or death due to ICD code H25
Cholelithiasis	History of gallstones during verbal interview with trained nurse or hospitalization for or death due to ICD code K56.3 or K80
Depression	History of depression during verbal interview with trained nurse or hospitalization for or death due to ICD code F32
Hypothyroidism	History of hypothyroidism during verbal interview with trained nurse or hospitalization for or death due to ICD code E03
Gastric reflux	History of gastric reflux during verbal interview with trained nurse or hospitalization for or death due to ICD code K21
Osteoporosis	History of osteoporosis during verbal interview with trained nurse or hospitalization for or death due to ICD code M80 or M81
Osteoarthritis	History of osteoarthritis during verbal interview with trained nurse or hospitalization for or death due to ICD code M15-19
Psoriasis	History of psoriasis during verbal interview with trained nurse or hospitalization for or death due to ICD code L40

Abbreviations: ICD, international classification of disease

Supplementary Table 8. Predicted loss of function variants in PDE3B identified in the Myocardial Infarction Genetics Consortium.

Variant	Effect of Variant	Carriers in Cases	Carriers in Controls
11:14666481_C/CTG	Frameshift: p.Ile289Ter	0	1
11:14666497_G/GAGGA	Frameshift: p.Arg294Lys	0	1
11:14810650_A/G	Splice Acceptor: c.1279-2A>G	0	1
11:14839865_AT/A	Frameshift: p.Ser554Leu	0	1
11:14840680_A/T	Splice Acceptor: c.1734-2A>T	1	0
11:14840732_CA/C	Frameshift: p.Asp596Ile	1	1
11:14854304_G_T	Stop Gained: p.Glu711Ter	0	1
11:14854367_C/T	Stop Gained: p.Arg732Ter	1	1
11:14865399_C/T	Stop Gained: p.Arg783Ter	8	15
11:14880600_C/A	Stop Gain: p.Tyr844Ter	1	0
11:14882913_G/T	Splice Donor: c.2886+1G>T	0	1

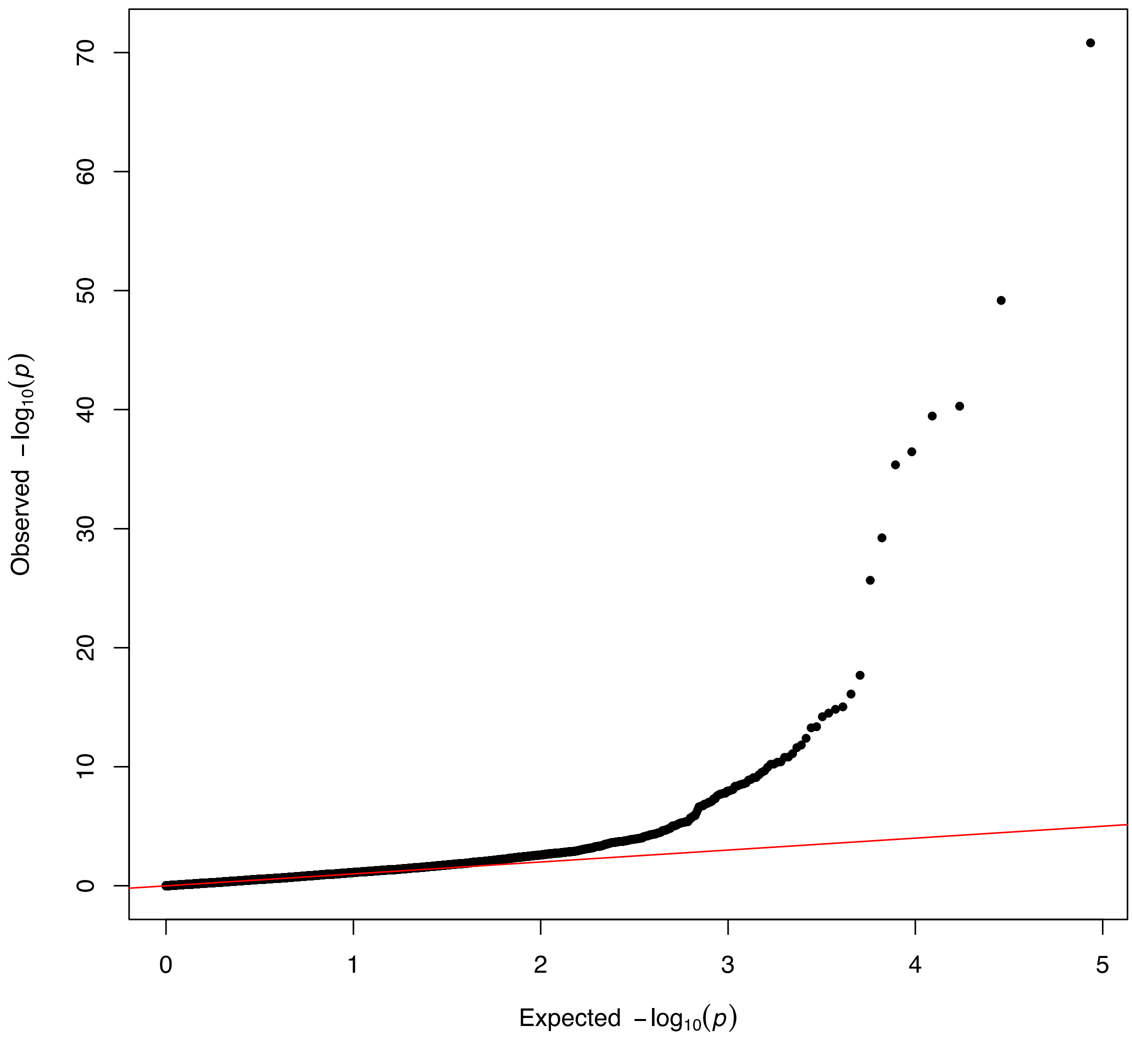

Supplementary Figure 1: Quantile-quantile plot for predicted loss-of-function analysis. No evidence of genomic inflation was observed (lambda = 1.09).

References

1. Igartua C, Myers RA, Mathias RA, et al. Ethnic-specific associations of rare and low-frequency DNA sequence variants with asthma. *Nat Commun*. 2015;6:5965. doi:10.1038/ncomms6965.

2. Locke AE, Kahali B, Berndt SI, et al. Genetic studies of body mass index yield new insights for obesity biology. *Nature*. 2015;518(7538):197-206. doi:10.1038/nature14177.

3. Khera AV, Won H-H, Peloso GM, et al. Association of Rare and Common Variation in the Lipoprotein Lipase Gene With Coronary Artery Disease. *JAMA : the journal of the American Medical Association*. 2017;317(9):937-946. doi:10.1001/jama.2017.0972.

4. Wood AR, Esko T, Yang J, et al. Defining the role of common variation in the genomic and biological architecture of adult human height. *Nat Genet*. 2014;46(11):1173-1186. doi:10.1038/ng.3097.

5. Eriksson N, Tung JY, Kiefer AK, et al. Novel associations for hypothyroidism include known autoimmune risk loci. Sawalha AH, ed. *PLoS ONE*. 2012;7(4):e34442. doi:10.1371/journal.pone.0034442.

6. Tsoi LC, Spain SL, Knight J, Ellinghaus E, Stuart PE. Identification of 15 new psoriasis susceptibility loci highlights the role of innate immunity. *Nature*. 2012.

7. Palmer CNA, Irvine AD, Terron-Kwiatkowski A, et al. Common loss-of-function variants of the epidermal barrier protein filaggrin are a major predisposing factor for atopic dermatitis. *Nat Genet*. 2006;38(4):441-446. doi:10.1038/ng1767.

8. Fernando MMA, Stevens CR, Walsh EC, et al. Defining the role of the MHC in autoimmunity: a review and pooled analysis. Fisher EMC, ed. *PLoS Genet*. 2008;4(4):e1000024. doi:10.1371/journal.pgen.1000024.

9. Miller AM. Role of IL-33 in inflammation and disease. *Journal of Inflammation*. 2011;8(1):22. doi:10.1186/1476-9255-8-22.

10. Broms J, Grahm M, Haugegaard L, Blom T, Meletis K, Tingström A. Monosynaptic retrograde tracing of neurons expressing the G-protein coupled receptor Gpr151 in the mouse brain. *J Comp Neurol*. 2017;525(15):3227-3250. doi:10.1002/cne.24273.

11. Hogan MC, Griffin MD, Rossetti S, Torres VE, Ward CJ, Harris PC. PKHDL1, a homolog of the autosomal recessive polycystic kidney disease gene, encodes a receptor with inducible T lymphocyte expression. *Hum Mol Genet*. 2003;12(6):685-698.

12. Reaux A, Iturrioz X, Vazeux G, et al. Aminopeptidase A, which generates one of

the main effector peptides of the brain renin-angiotensin system, angiotensin III, has a key role in central control of arterial blood pressure. *Biochem Soc Trans*. 2000;28(4):435-440.

13. Arnett HA, Viney JL. Immune modulation by butyrophilins. *Nat Rev Immunol*. 2014;14(8):559-569. doi:10.1038/nri3715.

14. Zhu M, Yan C, Ren C, et al. Exome Array Analysis Identifies Variants in SPOCD1 and BTN3A2 That Affect Risk for Gastric Cancer. *Gastroenterology*. 2017;152(8):2011-2021. doi:10.1053/j.gastro.2017.02.017.

15. Ampuero J, del Campo JA, Rojas L, et al. Fine-mapping butyrophilin family genes revealed several polymorphisms influencing viral genotype selection in hepatitis C infection. *Genes Immun*. 2015;16(5):297-300. doi:10.1038/gene.2015.14.

16. Kurima K, Yang Y, Sorber K, Griffith AJ. Characterization of the transmembrane channel-like (TMC) gene family: functional clues from hearing loss and epidermodysplasia verruciformis. *Genomics*. 2003;82(3):300-308.

17. Fawcett L, Baxendale R, Stacey P, et al. Molecular cloning and characterization of a distinct human phosphodiesterase gene family: PDE11A. *Proc Natl Acad Sci USA*. 2000;97(7):3702-3707. doi:10.1073/pnas.050585197.

18. Horvath A, Boikos S, Giatzakis C, et al. A genome-wide scan identifies mutations in the gene encoding phosphodiesterase 11A4 (PDE11A) in individuals with adrenocortical hyperplasia. *Nat Genet*. 2006;38(7):794-800. doi:10.1038/ng1809.

19. Gerding WM, Schreiber S, Schulte-Middelmann T, et al. Ccdc66 null mutation causes retinal degeneration and dysfunction. *Hum Mol Genet*. 2011;20(18):3620-3631. doi:10.1093/hmg/ddr282.

20. Deiss LP, Feinstein E, Berissi H, Cohen O, Kimchi A. Identification of a novel serine/threonine kinase and a novel 15-kD protein as potential mediators of the gamma interferon-induced cell death. *Genes Dev*. 1995;9(1):15-30.

21. Jia Y, Ye L, Ji K, et al. Death associated protein 1 is correlated with the clinical outcome of patients with colorectal cancer and has a role in the regulation of cell death. *Oncol Rep*. 2014;31(1):175-182. doi:10.3892/or.2013.2866.

22. Wazir U, Jiang WG, Sharma AK, Mokbel K. The mRNA expression of DAP1 in human breast cancer: correlation with clinicopathological parameters. *Cancer Genomics Proteomics*. 2012;9(4):199-201.

23. Noguchi K, Okumura F, Takahashi N, et al. TRIM40 promotes neddylation of IKKγ and is downregulated in gastrointestinal cancers. *Carcinogenesis*. 2011;32(7):995-1004. doi:10.1093/carcin/bgr068.

24. Bauer S, Groh V, Wu J, et al. Activation of NK cells and T cells by NKG2D, a

receptor for stress-inducible MICA. *Science*. 1999;285(5428):727-729.

25. Groh V, Rhinehart R, Randolph-Habecker J, Topp MS, Riddell SR, Spies T. Costimulation of CD8alphabeta T cells by NKG2D via engagement by MIC induced on virus-infected cells. *Nature immunology*. 2001;2(3):255-260. doi:10.1038/85321.

26. Hüe S, Mention J-J, Monteiro RC, et al. A direct role for NKG2D/MICA interaction in villous atrophy during celiac disease. *Immunity*. 2004;21(3):367-377. doi:10.1016/j.immuni.2004.06.018.

27. DiPilato LM, Ahmad F, Harms M, Seale P, Manganiello V, Birnbaum MJ. The Role of PDE3B Phosphorylation in the Inhibition of Lipolysis by Insulin. *Mol Cell Biol*. 2015;35(16):2752-2760. doi:10.1128/MCB.00422-15.

28. Ahmad F, Chung YW, Tang Y, et al. Phosphodiesterase 3B (PDE3B) regulates NLRP3 inflammasome in adipose tissue. *Sci Rep*. 2016;6(1):28056. doi:10.1038/srep28056.

29. Chung YW, Lagranha C, Chen Y, et al. Targeted disruption of PDE3B, but not PDE3A, protects murine heart from ischemia/reperfusion injury. *Proc Natl Acad Sci USA*. 2015;112(17):E2253-E2262. doi:10.1073/pnas.1416230112.

30. Tsuchikane E, Fukuhara A, Kobayashi T, et al. Impact of cilostazol on restenosis after percutaneous coronary balloon angioplasty. *Circulation*. 1999;100(1):21-26.

31. Regard JB, Scheek S, Borbiev T, et al. Verge: a novel vascular early response gene. *J Neurosci*. 2004;24(16):4092-4103. doi:10.1523/JNEUROSCI.4252-03.2004.

32. Rice GI, Del Toro Duany Y, Jenkinson EM, et al. Gain-of-function mutations in IFIH1 cause a spectrum of human disease phenotypes associated with upregulated type I interferon signaling. *Nat Genet*. 2014;46(5):503-509. doi:10.1038/ng.2933.

33. Stuart PE, Nair RP, Tsoi LC, et al. Genome-wide Association Analysis of Psoriatic Arthritis and Cutaneous Psoriasis Reveals Differences in Their Genetic Architecture. *Am J Hum Genet*. 2015;97(6):816-836. doi:10.1016/j.ajhg.2015.10.019.

34. Jin Y, Andersen G, Yorgov D, et al. Genome-wide association studies of autoimmune vitiligo identify 23 new risk loci and highlight key pathways and regulatory variants. *Nat Genet*. 2016;48(11):1418-1424. doi:10.1038/ng.3680.

35. Nejentsev S, Walker N, Riches D, Egholm M, Todd JA. Rare variants of IFIH1, a gene implicated in antiviral responses, protect against type 1 diabetes. *Science*. 2009;324(5925):387-389. doi:10.1126/science.1167728.

36. Chauhan S, Goodwin JG, Chauhan S, et al. ZKSCAN3 is a master transcriptional

repressor of autophagy. *Mol Cell*. 2013;50(1):16-28. doi:10.1016/j.molcel.2013.01.024.

37. Yang L, Hamilton SR, Sood A, et al. The previously undescribed ZKSCAN3 (ZNF306) is a novel “driver” of colorectal cancer progression. *Cancer Res*. 2008;68(11):4321-4330. doi:10.1158/0008-5472.CAN-08-0407.

38. Maguire J, Santoro T, Jensen P, Siebenlist U. Gem: an induced, immediate early protein belonging to the Ras family. *SCIENCE-NEW YORK* …. 1994.

39. Béguin P, Nagashima K, Gonoi T, et al. Regulation of Ca2+ channel expression at the cell surface by the small G-protein kir/Gem. *Nature*. 2001;411(6838):701-706. doi:10.1038/35079621.

40. Gautron S, Daegelen D, Mennecier F, Dubocq D, Kahn A, Dreyfus JC. Molecular mechanisms of McArdle's disease (muscle glycogen phosphorylase deficiency). RNA and DNA analysis. *J Clin Invest*. 1987;79(1):275-281. doi:10.1172/JCI112794.

41. Tsujino S, Shanske S, DiMauro S. Molecular genetic heterogeneity of myophosphorylase deficiency (McArdle's disease). *N Engl J Med*. 1993;329(4):241-245. doi:10.1056/NEJM199307223290404.

42. Myocardial Infarction Genetics and CARDIoGRAM Exome Consortia Investigators. Coding Variation in ANGPTL4, LPL, and SVEP1 and the Risk of Coronary Disease. *N Engl J Med*. 2016;374(12):1134-1144. doi:10.1056/NEJMoa1507652.
