# Supplementary material for: Analysis of predicted loss-of-function variants in UK Biobank identifies variants protective for disease
Source: Nat Commun. 2018 Apr 24;9:1613. doi: 10.1038/s41467-018-03911-8 (PMC5915445; doi:10.1038/s41467-018-03911-8)
Supplement: Supplementary file 2 — Description of Additional Supplementary Files [file 41467_2018_3911_MOESM2_ESM.pdf]

Description of Additional Supplementary Files

File Name: Supplementary Data 1
Description: Results of conditional analysis to identify independent variants at loci (+ 500 kb) where rare and low frequency loss of function variants associate with phenotypes in UK Biobank

File Name: Supplementary Data 2
Description: Results of conditional analysis to identify independent variants at loci (+ 500 kb) where common loss of function variants associate with phenotypes in UK Biobank.
